# Psychosis Associated With Hyperglycemia in a Female Patient With Type 1 Diabetes Mellitus: A Case Report

**DOI:** 10.7759/cureus.61597

**Published:** 2024-06-03

**Authors:** Sarah Garikana, Diane Mathew, Soojae Hollowell

**Affiliations:** 1 Psychiatry, Icahn School of Medicine at Mount Sinai, New York, USA; 2 Psychiatry, State University of New York Upstate Medical University, Syracuse, USA

**Keywords:** autoimmune disorders, type 1 diabetes mellitus, hyperglycemia, psychosis, schizophrenia

## Abstract

This paper presents the case of a 22-year-old woman who was hospitalized multiple times with episodes of psychosis co-occurring with hyperglycemia. Her psychosis was characterized by auditory hallucinations, visual hallucinations, and disorganized speech and behavior. The patient has a prior medical history of type 1 diabetes mellitus (T1DM) and Graves’ disease and was non-adherent to diabetic diet and medications. The patient is a Somalian refugee who moved to the United States (US) a year ago. We explore the relatively unique observation of hyperglycemia-induced psychosis in the patient, specifically in the context of autoimmune disorders. We also discuss some of the complexities associated with the cultural aspects of mental health and diabetes management in refugee communities and their implications in clinical practice.

## Introduction

The intricate relationship between diabetes and psychotic disorders is a very important area of research, essential for both clinical practitioners and public health experts. Prior studies have demonstrated that diabetes has a higher prevalence in people with psychotic disorders ​[1,2]​. Individuals with schizophrenia exhibit a two-to-five-fold heightened risk of developing diabetes compared to the general population ​[3]​. Recent works have further unveiled the presence of common susceptibility genes between schizophrenia and diabetes ​[4]​, evidencing potential genetic mechanisms underlying this intricate relationship. Type 1 diabetes mellitus (T1DM) can also impact the brain structure and function in young children due to increased glucose variability ​[5]​. 

Moreover, the use of antipsychotic medications has emerged as a salient factor in increasing the risk of diabetes among individuals with psychotic disorders ​[6]​. Despite the complex multifaceted nature of the relationship between diabetes and psychotic disorders, it is still unclear whether extreme fluctuations in blood sugar levels, such as hyperglycemia or hypoglycemia, could serve as causative triggers for psychotic episodes. Likewise, it is also unknown if a psychotic episode could have any impact on glucose regulation in the body. 

In this paper, we present the case of a 22-year-old woman with prior diagnoses of T1DM and Graves' disease, whose multiple hospital admissions were characterized by episodes of psychosis coinciding with hyperglycemia. To our knowledge, cases of such a nature are rarely reported in scholarly literature, thereby rendering this report of interest and value to the psychiatric community. Also of interest is the patient's status as a Somalian refugee, which provides an opportunity to explore the cultural dimensions of mental health and diabetes in the Somalian refugee community and their potential implications on the patient. 

## Case presentation

The patient, a female Somalian refugee, has a medical history of T1DM, Graves’ disease, and a familial history of postpartum hallucinations observed in her mother. She has been living in the US with her father and brothers for seven months prior to her first hospitalization. Her mother and sisters live in Somalia. She first presented to the emergency department with a headache and auditory hallucinations. Diagnostic evaluation revealed that her blood glucose level was 340, and she was in diabetic ketoacidosis (DKA) with an HbA1C of 8.1. She was appropriately treated for hyperglycemia. Her thyroid levels were also tested, but they were normal. Once stabilized, she was transferred to the psychiatric department for further assessment.

During her psychiatric interviews, she reported hearing voices, laughing, and also hearing the Quran. The patient often spoke incoherently during the interview, responding to her auditory hallucinations by saying, "Just get away from me, you are lying." She denied any suicidal ideations and visual hallucinations. She also reported that she did not recognize her brother, who was present in the room during the interview. We obtained collateral information from her father and brothers, which revealed disturbed sleep for three days prior to her hospital admission and also that she had been acting unusual for the last three weeks. They also reported incoherent speech, urinating in bed, and neglect of responsibilities such as cleaning, school, and work. Her brother mentioned that she was not depressed but was lonely as there were no other women in the house. The patient has no history of smoking, alcohol consumption, or seizures. 

The patient was diagnosed with unspecified schizophrenia spectrum and other psychotic disorders per DSM-5 ​[7]​. She was prescribed valproic acid 250 mg, haloperidol 5 mg, hydroxyzine 50 mg, and risperidone 2 mg. All prescriptions are specified on a daily basis. Her blood glucose levels would fluctuate because she was not fully adherent to her diabetic diet or medications. Her condition improved over the two weeks of hospitalization, and she was discharged. Her blood sugar at the time of discharge was 221. She was prescribed risperidone 2 mg along with her home medications, including atenolol 25 mg, glucagon 3 mg, glucose 4 gm, insulin lispro 100 unit/ml, Lantus SoloStar 100 unit/ml, melatonin 3 mg, methimazole 5 mg, omeprazole 40 mg, polyethylene glycol 17 gm, and vitamin D.

Ten days after discharge, she presented again to the hospital with visual hallucinations. Upon evaluation, the hospital found her to be hyperglycemic, with blood glucose levels at 389, and she was in diabetic ketoacidosis (DKA). Despite receiving appropriate treatment to resolve her DKA, she continued to experience visual hallucinations, including seeing bones protruding from the walls and seeing a "scary" version of herself with sharp teeth in the mirror. She also exhibited extreme agitation and psychosis; attempting to elope, being physically aggressive toward staff, and ripping decor off the walls, necessitating multiple doses of intramuscular haloperidol for sedation. Risperidone was restarted; her family reported that the patient had not been compliant with risperidone or insulin since discharge. The following medications were initiated: valproic acid 250 mg, Haldol 5 mg, hydroxyzine 50 mg, and Cogentin 1 mg.

She was also noted to be pacing in room, listening to music as she reported this calms her anxiety. While in the unit, she exhibited intrusive behavioral issues such as physical aggression and inappropriate sexual advances toward male patients in the ward. Additionally, she was preoccupied with finding a husband throughout her hospital admission. She often had to be placed on one-to-one status because she needed constant redirection. When staff members attempted to redirect her, she would become violent by hanging and grabbing other staff members. She was given Ativan, Haldol as needed, and Benadryl for agitation. 

She was discharged after one week as her glucose levels became stable and her condition improved. At the time of discharge, she reported to be in a "good mood" and her blood glucose levels were 210. There were no symptoms of depression, mania, anxiety, or psychosis. She was prescribed valproic acid 250 mg, risperidone (long-acting subcutaneous), and hydroxyzine 50 mg along with her home medications. 

One week later, the patient was presented to the hospital due to visual hallucinations and occasional stomach pains. She had visual hallucinations of seeing people in their skeletal forms when she was walking around. She did not have any suicidal or homicidal ideations. She had disorganized speech, internal preoccupation, and a flat affect. She was intrusive and hypersexual, which posed imminent harm, and therefore had to be secluded at times. The patient had akathisia on admission, and Ativan was given to provide relief. She was started on haloperidol 5 mg, which was later increased to 10 mg. She began showing improvement in her symptoms and gained some insight. The patient was discharged after seven weeks of hospitalization. She was prescribed additional valproic acid 500 mg nightly, Benztropine 2 mg, and hydroxyzine 50 mg along with her home medications. To be able to better control hyperglycemia on an outpatient basis, an insulin pump was obtained while the patient was inpatient, along with diabetes education. Her blood sugar was 158 at the time of discharge. She hasn't had any psychiatric admissions since discharge, and her endocrine department reports that she is managing her blood sugar levels well. The blood sugar levels observed during various stages are tabulated below in Table 1. All the medications prescribed at final discharge are summarized in Table 2. 

**Table 1:** Blood sugar levels of the patient at various times during the course of admissions and discharges.

Admissions and discharges	Blood sugar levels (mg/dL)
First admission	340
First discharge	221
Second admission	389
Second discharge	210
Third admission	320
Third discharge	158

**Table 2:** Discharge medications and their corresponding dosages.

Medication	Dosages and frequency
Valproic acid	125 mg, oral, twice daily and four nightly
Benztropine	1 mg, oral, twice daily
Hydroxyzine	50 mg, oral, nightly
Atenolol	12.5 mg, oral, daily
Glucagon	3 mg into a single nostril, if no response may repeat in 15 minutes
Glucose	16 g, oral, pro re nata (PRN)
Hydroxyzine	50 mg, oral, PRN
Insulin Lispro	100 unit/ml vial
Lantus SoloStar	100 unit/ml pen (In case of pump failure)
Melatonin	3 mg, oral, nightly
Methimazole	15 mg, oral, daily
Omeprazole	40 mg, oral, daily
Polyethylene glycol	17 gm, oral, daily
Vitamin D	1.25 mg (50,000 UT), oral, once every seven days

## Discussion

Our differential diagnoses for the patient included delirium, bipolar disorder with psychotic features, schizoaffective disorder, major depressive disorder with psychotic features, and schizophrenia. We ruled out delirium because the patient was oriented to time, place, and environment throughout her hospitalization, and there was no waxing and waning of consciousness. Bipolar disorder was eliminated due to the absence of mania, both historically and during hospitalization. Although the patient experienced sleep disturbances before her first admission, she did not exhibit insomnia or hypersomnia. Her family reported suicidal ideation, but the patient denied having any suicidal thoughts or being sad or depressed. Her cultural and religious beliefs, which discouraged suicide, along with instincts of self-preservation, family cohesion, and social integration, acted as protective factors.

During the course of hospitalization, her mood was mostly euthymic, and she did not meet the criteria for a depressive disorder diagnosis. The lack of any mood symptoms helped eliminate schizoaffective disorder. The patient exhibited auditory hallucinations, visual hallucinations, disorganized speech, and disorganized behavior, and her symptoms limited her functioning in self-care, work, and interpersonal relationships. These symptoms were seen throughout all her hospitalizations, which spanned over four months. The collateral information from her family revealed a prodromal phase of several months prior to her first hospitalization. Thus, we diagnosed her with schizophrenia per DSM-5 [7]. The DSM-5 diagnostic criteria for schizophrenia and the corresponding observations in the patient are summarized in Table 3. 

**Table 3:** DSM-5 diagnostic criteria for schizophrenia and the corresponding observations in the patient.

Diagnostic criteria	Observations in the patient
Characteristic symptoms	Visual hallucinations, auditory hallucinations, disorganized speech, disorganized behavior
Social/occupational dysfunction	Symptoms limited her functioning in self-care, work, and interpersonal relationships
Duration	Symptoms observed during hospitalizations (>4 months), prodromal phase of several months prior to hospitalization
Schizoaffective and mood disorder exclusion	No major depressive, manic, or mood episodes
Substance/general medical condition exclusion	No history of drug abuse, and symptoms are not attributable to another medical condition
Relationship to a pervasive developmental disorder	No autism spectrum disorder or a communication disorder of childhood onset

To our knowledge, there are only a few case reports that explicitly document psychotic symptoms associated with hyperglycemia, as in our patient. Sahoo et al. ​[8]​ presented a 36-year-old woman with T1DM who developed recurrent episodes of psychosis following poor adherence to her insulin therapy. Lopes et al. ​[9] reported a case of an 80-year-old woman with delirium and psychotic symptoms associated with hyperglycemia from poorly controlled type 2 diabetes mellitus (T2DM). On a related note, Bauer et al. ​[10]​ presented a case series of six patients who were diagnosed with T2DM after being admitted for acute psychosis. The exact mechanism that relates psychotic episodes to hyperglycemia is still unknown, highlighting the need for further research. Such evidence could prove critical in prompting clinicians to consider glycemic fluctuations as a potential cause of psychosis in diabetic patients. 

Our patient has a complex medical history with prior diagnoses of both Graves' disease and T1DM, highlighting the co-occurrence of two autoimmune disorders. Recent evidence suggests that patients with autoimmune thyroid dysfunction (AITD) have a high prevalence of other autoimmune conditions ​[11]​. Graves' disease exhibits a significant association with T1DM, particularly in female patients ​[12]​. T1DM and AITD, when occurring simultaneously, tend to have similar immunogenetic susceptibilities ​[13]​. Autoimmune disorders are also known to be significant risk factors for schizophrenia ​[14,15]​. These findings prompt us to consider if there is an underlying common immune dysregulation mechanism that is likely responsible for all the diseases in our patients ​[16]​.

Our patient belongs to a Somalian refugee community where significant stigma is associated with psychiatric disorders ​[17]​. Within these communities, psychological issues are often not openly acknowledged, and families may prefer to provide care within the home setting, with institutionalization considered a last resort ​[18]​. We observed a manifestation of this trend in our patient, particularly in her brother's characterization of her depression as stemming from loneliness due to the absence of other women in the household. We expect that our patient may have had psychiatric issues from a younger age, yet these issues may not have been fully recognized or addressed due to the prevailing stigma within Somalian refugee communities ​[19]​. These findings highlight the importance of cultural sensitivity in mental health care for refugee populations, where traditional beliefs and societal attitudes may be significantly different from Western culture.

Prior research ​[20]​ also reports lower levels of diabetes health literacy among Somalian refugee patients. These findings could be pertinent to our patient as she has repeatedly exhibited non-compliance with her diabetes medication and diet, particularly after relocating to the United States. We expect the patient could benefit from diabetes education, both for herself and her immediate family. Additionally, we also expect that an insulin pump could further help her with diabetes management, potentially reducing the recurrence of psychosis incidents.

## Conclusions

This paper presents observations of hyperglycemia-induced psychosis in a female patient with T1DM. This phenomenon is relatively under-reported in psychiatry literature and warrants further research to help identify underlying causative mechanisms, thus providing valuable guidance to clinicians. This case also underscores the importance of caution and guidance in treating psychosis in diabetic patients, as antipsychotic medications could further exacerbate their diabetes. 

Another intriguing aspect of this case is the co-occurrence of two autoimmune conditions alongside schizophrenia, which is also often expected to have an autoimmune basis. Therefore, we suspect the patient may have a common underlying immune dysregulation mechanism responsible for her various conditions. Lastly, this case highlights the importance of cultural sensitivity and awareness when treating patients from refugee communities. 
